# Differential Development of Dendritic Spines in Striatal Projection Neurons of Direct and Indirect Pathways in the Caudoputamen and Nucleus Accumbens

**DOI:** 10.1523/ENEURO.0366-22.2023

**Published:** 2023-06-09

**Authors:** Hsiao-Ying Kuo, Ya-Hui Yang, Shih-Yun Chen, Tzu-Hsin Kuo, Wan-Ting Lin, Fu-Chin Liu

**Affiliations:** 1 Institute of Anatomy and Cell Biology, National Yang Ming Chiao Tung University, Taipei 112304, Taiwan; 2 Institute of Neuroscience, National Yang Ming Chiao Tung University, Taipei 112304, Taiwan

**Keywords:** basal ganglia, spine formation, spine pruning, spinogenesis, striatum, synaptogenesis

## Abstract

Synaptic modification in postnatal development is essential for the maturation of neural networks. Developmental maturation of excitatory synapses occurs at the loci of dendritic spines that are dynamically regulated by growth and pruning. Striatal spiny projection neurons (SPNs) receive excitatory input from the cerebral cortex and thalamus. SPNs of the striatonigral direct pathway (dSPNs) and SPNs of the striatopallidal indirect pathway (iSPNs) have different developmental roots and functions. The spatial and temporal dynamics of dendritic spine maturation of these two types of SPNs remain elusive. Here, we delineate the developmental trajectories of dendritic spines of dSPNs and iSPNs in the caudoputamen and nucleus accumbens (NAc). We labeled dendritic spines of SPNs by microinjecting Cre-dependent AAV-eYFP viruses into newborn Drd1-Cre or Adora2a-Cre mice, and analyzed spinogenesis at three levels, including different SPN cell types, subregions and postnatal times. In the dorsolateral striatum, spine pruning of dSPNs and iSPNs occurred at postnatal day (P)30–P50. In the dorsomedial striatum, the spine density of both dSPNs and iSPNs reached its peak between P30 and P50, and spine pruning occurred after P30 and P50, respectively, for dSPNs and iSPNs. In the NAc shell, spines of dSPNs and iSPNs were pruned after P21–P30, but no significant pruning was observed in iSPNs of lateral NAc shell. In the NAc core, the spine density of dSPNs and iSPNs reached its peak at P21 and P30, respectively, and subsequently declined. Collectively, the developmental maturation of dendritic spines in dSPNs and iSPNs follows distinct spatiotemporal trajectories in the dorsal and ventral striatum.

## Significance Statement

The direct striatonigral and indirect striatopallidal pathways are engaged in neural circuits of basal ganglia for the regulation of movement and drug addiction. Such circuit functions rely on precise synaptic connectivity that goes through the maturation process. Excitatory synaptic connectivity can be traced by examining the development of dendritic spines. Here, we provide a comprehensive characterization of the development of dendritic spines in spiny projection neurons (SPNs) of the direct and indirect pathways from juvenile and adolescent to adult stages in the mouse brain. We found distinct cell type-specific trajectories of dendritic spines in the caudoputamen and nucleus accumbens (NAc). Our study provides a basic reference for neuropsychiatric diseases in which dysfunction of spinogenesis and synaptogenesis is targeted during development, including autism and schizophrenia.

## Introduction

The basal ganglia are a group of subcortical nuclei serving a variety of neurologic functions, including motor control, reward learning, motivation, and emotion (Graybiel and Grafton, 2015; Keiflin and Janak, 2015; Kim and Hikosaka, 2015; Pessoa et al., 2019). The striatum is the main input structure of the basal ganglia. The striatum consists of two populations of projection neurons: dopamine D1 receptor-expressing striatonigral projection neurons of the direct pathway (dSPNs) and dopamine D2 receptor-expressing striatopallidal projection neurons of the indirect pathway (iSPNs; Gerfen et al., 1990; Gerfen and Surmeier, 2011). The prevailing theory suggests that activation of the direct pathway facilitates movement, whereas activation of the indirect pathway inhibits movement (Albin et al., 1989; Gerfen et al., 1990; Kravitz et al., 2010). The coordination of neuronal activity in direct and indirect pathways is thus important for movement control (Macpherson and Hikida, 2019; Arber and Costa, 2022).

The striatal complex is divided into the dorsal striatum (caudoputamen) and the ventral striatum [nucleus accumbens (NAc); Haber, 2016; Chen et al., 2020]. The caudoputamen is involved in the regulation of movement, motor learning, habits and decision-making (Mink, 2003; Gittis and Kreitzer, 2012; DeLong and Wichmann, 2015; Gunaydin and Kreitzer, 2016; Macpherson and Hikida, 2019). The nucleus accumbens is a key hub of reward circuits that are engaged in processing reward, motivation, emotion and drug addiction (Volkow et al., 2012; Russo and Nestler, 2013; Klawonn and Malenka, 2018; Nestler and Lüscher, 2019). The NAc is divided into the core and shell regions, and they have distinct neuronal connectivity that enables specific functions (West and Carelli, 2016; Li et al., 2018; Yang et al., 2018; Ma et al., 2020).

The neuronal architecture is specialized for neural processing in networks (Parekh and Ascoli, 2015). Dendritic spines are small membrane protrusions and specialized cell compartments through which neurons receive excitatory input from presynaptic axonal terminals. Dynamic changes in dendritic spine size, density and morphologic types occur not only in neuronal development but also in neuronal plasticity such as learning and memory (Segal, 2017; Runge et al., 2020). Abnormality in dendritic spine formation has frequently been found in neuropsychiatric diseases, including depression, schizophrenia and drug addiction (Roberts et al., 1996; Russo et al., 2010; Francis et al., 2017).

Synaptogenesis occurs after axonal outgrowth to innervate target cells during development. Activity-dependent modification of synaptogenesis further refines synaptic connectivity through synapse pruning (Südhof, 2018; Batool et al., 2019). Dendritic spine pruning plays an important role in the formation of mature excitatory synaptic connectivity, since the synaptic strength of neurotransmission is influenced by the density of spines/synapses (Carlisle and Kennedy, 2005; Ding et al., 2011; Grueter et al., 2012; Sala and Segal, 2014). Dendritic spines undergo plastic changes in response to environmental stimuli. The prevailing theory suggests that in the developing mammalian brains, dendritic spines initially overgrowth, followed by pruning during juvenile and adolescent stages before maturation in adulthood (Segal et al., 2000; Semple et al., 2013). Importantly, defective synaptic pruning in postnatal brains has been implicated in the pathogenesis of neuropsychiatric diseases (Hutsler and Zhang, 2010; Forrest et al., 2018; Lima Caldeira et al., 2019; Eltokhi et al., 2020), which calls for the understanding of synaptic modification during postnatal maturation. Previous studies have reported the development process of spinogenesis in the cerebellum, cerebral cortex, and hippocampus (García-López et al., 2010; Elston and Fujita, 2014). However, the developmental trajectory of spinogenesis of striatal neurons remains yet elusive.

In the present study, we investigated the developmental trajectory of dendritic spinogenesis of dSPNs and iSPNs in the caudoputamen and NAc. We found distinct trajectories of spinogenesis of dSPNs and iSPNs in subregions of the striatum during postnatal development, suggesting that synaptic wiring in striatal subregions is under different spatiotemporal control.

## Materials and Methods

### Animals

All animals were housed in groups in a 12/12 h light/dark cycle-specific pathogen-free room with food and water available *ad libitum* at the Animal Center of National Yang Ming Chiao Tung University. All experimental procedures in this study were approved by the Institutional Animal Care and Use Committee of National Yang Ming Chiao Tung University. Dopamine receptor D1 (Drd1)-Cre mice (STOCK Tg(Drd1-cre)EY262Gsat/Mmucd, RRID: MMRRC_017264-UCD) and adenosine receptor 2a (Adora2a)-Cre mice (STOCK Tg(Adora2a-cre)KG139Gsat/Mmucd, RRID: MMRRC_031168-UCD) were obtained from Mutant Mouse Resource and Research Centers supported by the National Institutes of Health. *Drd1-Cre* and *Adora2a-Cre* mice were maintained by intercrossing with wild-type C57BL/6J mice. Male mice were used in the current study, because gender-specific regulation of SPN maturation by sex hormones has previously been reported in rodents (Cao et al., 2018).

### Genotyping

Genotypes of the mice were identified at postnatal day (P)0 by PCR with genomic DNA. For DNA extraction, ∼0.2 mm of tail tissue was heated and dissolved in 25 mm NaOH/0.2 mm EDTA for 10 min at 100°C. The dissolved tissue was then neutralized with an equal volume of 40 mm Tris-EDTA buffer (pH5.5) and cooled on ice. The PCR protocols were as follows: 95°C for 3 min, 31 cycles at 95°C for 30 s, 58°C for 30 s, 72°C for 45 s with a thermocycler (T3000, Biometra). The reaction was completed at 72°C for an additional 5 min and paused at 4°C. The primers used to identify the Cre sequences are 5′-ATGCTTCTGTCCGTTTGCCG-3′ and 5′-TGAGTGAACGAACCTGGTCG-3′. The expected size of the product was 316 bp.

### Stereotaxic microinjections

Cre-dependent *AAV9.EF1α.DIO.eYFP.WPRE.hGH* (UPenn vector core; catalog #V-9-27056; lot #CS0977) viruses were diluted at 1:100 in D-PBS (CORNING-cellgro) for microinjections. The mouse pups (P0–P2) were anesthetized by hypothermia. *AAV9.EF1α.DIO.eYFP.WPRE.hGH* viruses (50 nl/site) were microinjected into the bilateral dorsal striatum (AP: +2.8 mm, ML: ±1.5 mm, DV: 1.5 mm) or ventral (AP: +2.8 mm, ML: ±1.0 mm, DV: 1.7 mm) striatum of *Drd1-Cre* or *Adora2a-Cre* mice.

### Tissue preparation and immunohistochemistry

The brains of *Drd1-Cre* and *Adora2a-Cre* mice were harvested at P13, P21, P30, P50, and P100 by transcardial perfusion of 0.9% saline followed by 4% paraformaldehyde (PFA) in 0.1 m PBS (pH 7.4). The perfused brains were postfixed in 4% PFA/0.1 m PBS at 4°C overnight and were then cryoprotected with 30% sucrose/0.1 m PBS for 48 h. The brains were cut into 80-μm coronal sections with a vibratome (D.S.K., DTK-1000) and stored in 0.1 m phosphate buffer (PB) with 0.1% sodium azide at 4°C. Immunohistochemistry was performed as previously described (Chen et al., 2016). Briefly, after permeabilization with 0.2% Triton X-100/0.1 m PBS and removal of endogenous peroxidase with 10% methanol/3% H_2_O_2_/0.2% Triton X-100/0.1 m PBS, brain sections were blocked with 3% normal donkey serum/0.1 m PBS for 1 h at room temperature. After rinsing with 0.1 m PBS, brain sections were incubated in chicken anti-GFP primary antibody (1:1000; Abcam catalog #ab13970, RRID: AB_300798) at 4°C overnight. The next day, brain sections were incubated with the biotinylated secondary antibody donkey anti-chicken (1:500, Vector Laboratories) for 1 h, followed by the avidin-biotin-peroxidase complex (1:250, Vector catalog #PK-6100, RRID: AB_2336819) for another 1 h. The sections were then developed in 0.02% diaminobenzidine/0.08% nickel ammonium sulfate/0.003% H_2_O_2_ in 0.1 m PB.

### Microscope imaging and quantification

Z-stack images of dendritic spines with 0.42 μm step size were acquired using an Olympus BX63 microscope with a 100× oil immersion objective. Neurons filled with eYFP signals were imaged. All imaged neurons were analyzed, except that the neurites of the imaged neurons were intermingled with the neurites of neighboring neurons. Images of eYFP-positive neurons were taken from the dorsal striatum at Bregma levels +1.7 to +0.14. A vertical line was drawn in the middle striatum to separate the dorsomedial and dorsolateral striatum. For the ventral striatum at Bregma +1.7 to +0.74, the core and shell of the NAc were identified by the gradient of cell density under a microscope. The medial and lateral parts of the NAc were divided by a vertical line from the middle part of the anterior commissure. The numbers of dendritic spines were manually counted in the proximal pars of the secondary dendrites and normalized to the lengths of the analyzed dendrites. Dendritic spine density was calculated as the average number of spines per 10 μm. We categorized the morphology of dendritic spines into six types according to Harris et al., with slight modifications (Harris et al., 1992). Spines were categorized as “stubby spines” if the diameter of the neck was similar to the length of the spine. Spines were categorized as “thin/filopodial spines” if the length of the spine was longer than the diameter of the neck; and the diameters of the head and neck were similar with a ratio is no more than two. Spines were categorized as “mushroom spines” if the diameter of the head was >2.5-fold of the diameter of the neck. Spines were categorized as “branched spines” and “multibranched spines” if they had two and more than two heads, respectively. The spines that could not be classified into a specific group were assigned to the category of “atypical spines.”

### Statistical analysis

Statistical analysis was performed with SPSS (IBM, version 21). First, all the data were tested for normality with Shapiro–Wilk test. All data in this study were consistent with a normal distribution and we used two-way ANOVA to examine the influences of developmental times and genotypes on dendritic spine density. In the event of no interaction, one-way ANOVA followed by Tukey’s HSD *post hoc* tests was used to analyze the effects of developmental times in each genotype. If there was an interaction, simple main effects were performed to identify significant differences between developmental times. To analyze the temporal changes of each type of spine, one-way ANOVA followed by Tukey’s HSD *post hoc* tests were used for normally distributed data. For the data that are not normally distributed, Kruskal–Wallis one-way ANOVA followed by Dunn’s pairwise multiple comparisons tests or Mann–Whitney *U* test was used. To analyze the differences between the dorsomedial and dorsolateral striatum at each developmental time point, Student’s *t* tests were used. To analyze the differences among the NAc subregions at each developmental time point, one-way ANOVA followed by Tukey’s HSD *post hoc* tests were used. Data were presented as mean ± SEM if the data were normally distributed. Data were presented as median ± interquartile range if the data were analyzed by nonparametric analysis. Table 1 shows the details of statistical analyses.

**Table 1:** Statistical analyses

	Data structure	Type of test	95% confidence interval	
			Lower bound	Upper bound
Figure 1*D*, dSPN				
P13	Normal distribution	Two-way ANOVA	4.713	8.103
P21	Normal distribution	Two-way ANOVA	7.393	10.782
P30	Normal distribution	Two-way ANOVA	12.956	16.345
P50	Normal distribution	Two-way ANOVA	9.656	13.046
P100	Normal distribution	Two-way ANOVA	8.951	13.341
Figure 1*D*, iSPN				
P13	Normal distribution	Two-way ANOVA	8.062	11.452
P21	Normal distribution	Two-way ANOVA	9.312	12.701
P30	Normal distribution	Two-way ANOVA	15.355	18.744
P50	Normal distribution	Two-way ANOVA	11.748	15.137
P100	Normal distribution	Two-way ANOVA	7.623	11.012
Figure 1*F*, dSPN				
Stubby				
P13	Normal distribution	Kruskal–Wallis test	36.742	53.250
P21	Normal distribution		38.707	50.243
P30	Normal distribution		28.789	43.423
P50	Normal distribution		27.797	41.213
P100	Non-normal distribution		31.236	39.342
Thin/Filopodial				
P13	Normal distribution	One-way ANOVA	34.391	45.181
P21	Normal distribution		36.123	48.871
P30	Normal distribution		44.159	54.677
P50	Normal distribution		42.864	57.136
P100	Normal distribution		35.728	43.648
Mushroom				
P13	Non-normal distribution	Kruskal–Wallis test	4.471	16.927
P21	Normal distribution		7.168	12.234
P30	Normal distribution		7.523	14.089
P50	Normal distribution		6.555	14.385
P100	Normal distribution		16.950	25.212
Branched				
P13	Non-normal distribution	Kruskal–Wallis test	−0.184	8.220
P21	Non-normal distribution		0.326	5.594
P30	Non-normal distribution		0.261	5.791
P50	Normal distribution		1.996	7.238
P100	Non-normal distribution		2.080	5.166
Multibranched				
P13	Not applicable	Kruskal–Wallis test	Not applicable	
P21	Not applicable		Not applicable	
P30	Non-normal distribution		−0.240	0.620
P50	Non-normal distribution		−0.515	1.331
P100	Non-normal distribution		−0.170	0.814
Atypical				
P13	Not applicable	Mann–Whitney *U*	Not applicable	
P21	Non-normal distribution		−0.467	1.207
P30	Non-normal distribution		−0.574	1.484
P50	Not applicable		Not applicable	
P100	Not applicable		Not applicable	
Figure 1*F*, iSPN				
Stubby				
P13	Non-normal distribution	Kruskal–Wallis test	29.847	40.033
P21	Normal distribution		39.921	47.440
P30	Normal distribution		38.523	48.398
P50	Normal distribution		30.236	40.799
P100	Normal distribution		33.705	40.828
Thin/Filopodial				
P13	Normal distribution	One-way ANOVA	37.272	51.416
P21	Normal distribution		37.240	44.236
P30	Normal distribution		37.849	44.907
P50	Normal distribution		43.051	45.783
P100	Normal distribution		38.402	46.334
Mushroom				
P13	Non-normal distribution	Kruskal–Wallis test	6.244	28.854
P21	Normal distribution		9.035	16.238
P30	Normal distribution		7.531	14.029
P50	Normal distribution		11.163	17.228
P100	Normal distribution		11.965	19.652
Branched				
P13	Non-normal distribution	Kruskal–Wallis test	0.655	5.430
P21	Normal distribution		1.023	4.459
P30	Normal distribution		1.844	6.378
P50	Normal distribution		3.338	7.656
P100	Normal distribution		2.466	4.936
Multibranched				
P13	Not applicable	Kruskal–Wallis test	Not applicable	
P21	Non-normal distribution		−0.118	0.525
P30	Non-normal distribution		−0.341	0.882
P50	Non-normal distribution		−0.050	0.572
P100	Non-normal distribution		−0.004	1.459
Atypical				
P13	Non-normal distribution	Kruskal–Wallis test	−0.158	0.408
P21	Not applicable		Not applicable	
P30	Not applicable		Not applicable	
P50	Non-normal distribution		−0.142	0.367
P100	Non-normal distribution		−0.162	0.418
Figure 2*D*, dSPN				
P13	Normal distribution	Two-way ANOVA	5.139	8.236
P21	Normal distribution	Two-way ANOVA	8.373	11.469
P30	Normal distribution	Two-way ANOVA	10.991	14.087
P50	Normal distribution	Two-way ANOVA	9.548	12.645
P100	Normal distribution	Two-way ANOVA	8.567	11.663
Figure 2*D*, iSPN				
P13	Normal distribution	Two-way ANOVA	7.237	10.334
P21	Normal distribution	Two-way ANOVA	9.057	12.154
P30	Normal distribution	Two-way ANOVA	11.254	14.351
P50	Normal distribution	Two-way ANOVA	13.300	16.396
P100	Normal distribution	Two-way ANOVA	7.192	10.289
Figure 2*F*, dSPN				
Stubby				
P13	Normal distribution	One-way ANOVA	33.063	46.911
P21	Normal distribution		35.711	49.029
P30	Normal distribution		35.143	42.312
P50	Normal distribution		28.874	40.205
P100	Normal distribution		30.579	39.438
Thin/Filopodial				
P13	Normal distribution	One-way ANOVA	37.429	49.957
P21	Normal distribution		41.409	54.360
P30	Normal distribution		41.981	49.121
P50	Normal distribution		44.564	53.695
P100	Normal distribution		39.988	45.904
Mushroom				
P13	Normal distribution	Kruskal–Wallis test	9.872	18.020
P21	Normal distribution		3.334	9.160
P30	Normal distribution		8.098	15.757
P50	Non-normal distribution		7.214	13.084
P100	Normal distribution		13.042	22.791
Branched				
P13	Non-normal distribution	Kruskal–Wallis test	−0.301	3.987
P21	Non-normal distribution		0.249	5.323
P30	Normal distribution		1.342	4.814
P50	Non-normal distribution		3.301	8.600
P100	Normal distribution		2.381	5.878
Multibranched				
P13	Non-normal distribution	Kruskal–Wallis test	−0.227	1.008
P21	Non-normal distribution		−0.394	1.819
P30	Non-normal distribution		−0.157	0.731
P50	Non-normal distribution		−0.118	0.580
P100	Not applicable		Not applicable	
Atypical				
P13	Non-normal distribution	Mann–Whitney *U*	−0.178	0.460
P21	Not applicable		Not applicable	
P30	Non-normal distribution		−0.233	1.091
P50	Not applicable		Not applicable	
P100	Not applicable		Not applicable	
Figure 2*F*, iSPN				
Stubby				
P13	Normal distribution	One-way ANOVA	31.928	49.053
P21	Normal distribution		31.945	42.072
P30	Normal distribution		36.102	47.205
P50	Normal distribution		30.633	41.191
P100	Normal distribution		32.026	42.913
Thin/Filopodial				
P13	Normal distribution	One-way ANOVA	37.451	51.261
P21	Normal distribution		42.865	52.515
P30	Normal distribution		37.090	50.568
P50	Normal distribution		29.703	48.579
P100	Normal distribution		40.278	49.134
Mushroom				
P13	Normal distribution	Kruskal–Wallis test	6.817	17.795
P21	Non-normal distribution		7.399	18.539
P30	Normal distribution		8.693	19.137
P50	Normal distribution		13.486	26.657
P100	Non-normal distribution		10.660	16.904
Branched				
P13	Non-normal distribution	Kruskal–Wallis test	0.390	3.971
P21	Non-normal distribution		0.380	3.682
P30	Non-normal distribution		0.058	3.146
P50	Normal distribution		2.054	7.134
P100	Normal distribution		2.139	5.769
Multibranched				
P13	Non-normal distribution	Kruskal–Wallis test	−0.631	1.631
P21	Non-normal distribution		−0.187	0.792
P30	Not applicable		Not applicable	
P50	Non-normal distribution		−0.178	0.460
P100	Non-normal distribution		−0.112	0.289
Atypical				
P13	Non-normal distribution	Mann–Whitney *U*	−0.210	0.544
P21	Not applicable		Not applicable	
P30	Not applicable		Not applicable	
P50	Non-normal distribution		−0.178	0.460
P100	Not applicable		Not applicable	
Figure 3*D*, dSPN				
P13	Normal distribution	Two-way ANOVA	8.769	11.674
P21	Normal distribution	Two-way ANOVA	8.813	11.717
P30	Normal distribution	Two-way ANOVA	10.400	13.305
P50	Normal distribution	Two-way ANOVA	7.344	10.249
P100	Normal distribution	Two-way ANOVA	7.008	9.913
Figure 3*D*, iSPN				
P13	Normal distribution	Two-way ANOVA	7.340	10.245
P21	Normal distribution	Two-way ANOVA	6.423	9.328
P30	Normal distribution	Two-way ANOVA	7.042	9.947
P50	Normal distribution	Two-way ANOVA	8.567	11.472
P100	Normal distribution	Two-way ANOVA	7.165	10.070
Figure 3*F*, dSPN				
Stubby				
P13	Normal distribution	One-way ANOVA	35.886	46.900
P21	Normal distribution		37.407	50.410
P30	Normal distribution		35.555	46.824
P50	Normal distribution		29.784	40.883
P100	Normal distribution		25.271	33.411
Thin/Filopodial				
P13	Normal distribution	One-way ANOVA	36.756	48.498
P21	Normal distribution		35.852	48.491
P30	Normal distribution		31.125	41.900
P50	Normal distribution		36.678	47.183
P100	Normal distribution		47.323	50.933
Mushroom				
P13	Normal distribution	One-way ANOVA	6.090	11.397
P21	Normal distribution		7.172	16.549
P30	Normal distribution		13.375	23.500
P50	Normal distribution		15.079	24.542
P100	Normal distribution		14.252	18.435
Branched				
P13	Normal distribution	Kruskal–Wallis test	3.497	8.541
P21	Non-normal distribution		0.039	2.728
P30	Normal distribution		1.777	4.880
P50	Normal distribution		1.181	4.671
P100	Non-normal distribution		2.544	6.045
Multibranched				
P13	Normal distribution	Kruskal–Wallis test	0.314	1.658
P21	Non-normal distribution		−0.048	1.399
P30	Non-normal distribution		−0.163	0.804
P50	Not applicable		Not applicable	
P100	Non-normal distribution		−0.152	1.939
Atypical				
P13	Non-normal distribution	Mann–Whitney *U*	−0.294	0.759
P21	Not applicable		Not applicable	
P30	Non-normal distribution		−0.114	0.538
P50	Not applicable		Not applicable	
P100	Not applicable		Not applicable	
Figure 3*F*, iSPN				
Stubby				
P13	Normal distribution	One-way ANOVA	33.192	48.068
P21	Normal distribution		34.031	41.462
P30	Normal distribution		28.308	42.659
P50	Normal distribution		36.953	40.510
P100	Normal distribution		31.264	38.340
Thin/Filopodial				
P13	Normal distribution	One-way ANOVA	31.176	46.244
P21	Normal distribution		43.619	50.504
P30	Normal distribution		35.150	46.630
P50	Normal distribution		34.313	41.881
P100	Normal distribution		38.587	43.987
Mushroom				
P13	Normal distribution	One-way ANOVA	10.895	19.485
P21	Normal distribution		8.370	15.747
P30	Normal distribution		11.283	22.671
P50	Normal distribution		14.385	22.627
P100	Normal distribution		18.163	24.865
Branched				
P13	Normal distribution	Kruskal–Wallis test	2.889	6.931
P21	Normal distribution		1.528	4.401
P30	Non-normal distribution		1.394	11.906
P50	Normal distribution		2.690	5.878
P100	Normal distribution		0.839	2.958
Multibranched				
P13	Not applicable	Mann–Whitney *U*	Not applicable	
P21	Not applicable		Not applicable	
P30	Not applicable		Not applicable	
P50	Non-normal distribution		−0.221	0.983
P100	Non-normal distribution		−0.120	1.117
Atypical				
P13	Non-normal distribution	Mann–Whitney *U*	−0.101	1.222
P21	Non-normal distribution		−0.214	0.553
P30	Not applicable		Not applicable	
P50	Not applicable		Not applicable	
P100	Not applicable		Not applicable	
Figure 4*D*, dSPN				
P13	Normal distribution	Two-way ANOVA	7.472	10.088
P21	Normal distribution	Two-way ANOVA	8.464	11.079
P30	Normal distribution	Two-way ANOVA	12.942	15.557
P50	Normal distribution	Two-way ANOVA	9.576	12.191
P100	Normal distribution	Two-way ANOVA	9.615	12.231
Figure 4*D*, iSPN				
P13	Normal distribution	Two-way ANOVA	8.194	10.809
P21	Normal distribution	Two-way ANOVA	9.231	11.846
P30	Normal distribution	Two-way ANOVA	10.682	13.297
P50	Normal distribution	Two-way ANOVA	9.553	12.168
P100	Normal distribution	Two-way ANOVA	8.374	10.989
Figure 4*F*, dSPN				
Stubby				
P13	Normal distribution	One-way ANOVA	33.850	41.769
P21	Normal distribution		41.638	46.655
P30	Normal distribution		37.210	48.537
P50	Normal distribution		32.869	46.236
P100	Normal distribution		27.686	40.016
Thin/Filopodial				
P13	Normal distribution	One-way ANOVA	42.065	48.362
P21	Normal distribution		37.953	45.448
P30	Normal distribution		33.192	41.123
P50	Normal distribution		36.004	48.050
P100	Normal distribution		34.177	42.528
Mushroom				
P13	Normal distribution	One-way ANOVA	8.380	15.331
P21	Normal distribution		8.463	13.480
P30	Normal distribution		10.926	22.004
P50	Normal distribution		11.574	19.644
P100	Normal distribution		16.809	29.022
Branched				
P13	Normal distribution	One-way ANOVA	2.906	6.428
P21	Normal distribution		1.359	4.719
P30	Normal distribution		1.509	5.285
P50	Normal distribution		0.578	4.441
P100	Normal distribution		2.127	6.565
Multibranched				
P13	Non-normal distribution	Kruskal–Wallis test	−0.130	0.940
P21	Non-normal distribution		−0.180	0.466
P30	Non-normal distribution		−0.136	0.351
P50	Non-normal distribution		−0.160	0.413
P100	Non-normal distribution		−0.676	1.748
Atypical				
P13	Not applicable	Not applicable	Not applicable	
P21	Not applicable		Not applicable	
P30	Not applicable		Not applicable	
P50	Non-normal distribution		−0.221	0.572
P100	Not applicable		Not applicable	
Figure 4*F*, iSPN				
Stubby				
P13	Normal distribution	One-way ANOVA	39.285	46.048
P21	Normal distribution		39.255	48.969
P30	Normal distribution		36.944	43.832
P50	Normal distribution		35.812	45.406
P100	Normal distribution		36.527	41.910
Thin/Filopodial				
P13	Normal distribution	Kruskal–Wallis test	35.846	41.869
P21	Normal distribution		35.167	45.447
P30	Normal distribution		35.582	43.339
P50	Non-normal distribution		36.507	43.133
P100	Normal distribution		35.375	42.509
Mushroom				
P13	Normal distribution	One-way ANOVA	12.688	19.001
P21	Normal distribution		10.056	13.477
P30	Normal distribution		13.213	18.533
P50	Normal distribution		13.252	21.245
P100	Normal distribution		15.118	22.548
Branched				
P13	Normal distribution	One-way ANOVA	0.855	4.189
P21	Normal distribution		1.312	5.933
P30	Normal distribution		2.452	5.705
P50	Normal distribution		0.907	3.740
P100	Normal distribution		1.152	4.363
Multibranched				
P13	Non-normal distribution	Kruskal–Wallis test	−0.139	0.359
P21	Non-normal distribution		−0.243	0.627
P30	Non-normal distribution		−0.104	0.505
P50				
P100	Non-normal distribution		−0.126	0.623
Atypical				
P13	Not applicable	Not applicable	Not applicable	
P21	Not applicable	Not applicable		
P30	Not applicable	Not applicable		
P50	Not applicable	Not applicable		
P100	Not applicable	Not applicable		
Figure 5*D*, dSPN				
P13	Normal distribution	Two-way ANOVA	6.917	9.796
P21	Normal distribution	Two-way ANOVA	10.298	12.574
P30	Normal distribution	Two-way ANOVA	8.920	11.798
P50	Normal distribution	Two-way ANOVA	8.629	11.508
P100	Normal distribution	Two-way ANOVA	7.408	9.758
Figure 5*D*, iSPN				
P13	Normal distribution	Two-way ANOVA	7.217	10.096
P21	Normal distribution	Two-way ANOVA	7.404	10.283
P30	Normal distribution	Two-way ANOVA	9.421	12.299
P50	Normal distribution	Two-way ANOVA	9.540	12.418
P100	Normal distribution	Two-way ANOVA	7.003	9.353
Figure 5*F*, dSPN				
Stubby				
P13	Normal distribution	One-way ANOVA	35.906	43.439
P21	Normal distribution		36.294	47.263
P30	Normal distribution		27.286	43.385
P50	Normal distribution		28.076	40.691
P100	Normal distribution		29.939	38.538
Thin/Filopodial				
P13	Normal distribution	One-way ANOVA	40.338	51.234
P21	Normal distribution		39.407	47.054
P30	Normal distribution		42.124	54.845
P50	Normal distribution		36.213	48.237
P100	Normal distribution		35.388	41.348
Mushroom				
P13	Normal distribution	One-way ANOVA	9.094	13.298
P21	Normal distribution		7.943	15.476
P30	Normal distribution		7.968	16.939
P50	Normal distribution		13.707	23.974
P100	Normal distribution		19.883	27.036
Branched				
P13	Normal distribution	Kruskal–Wallis test	0.842	4.111
P21	Normal distribution		1.277	3.529
P30	Non-normal distribution		−0.936	5.831
P50	Normal distribution		1.767	7.091
P100	Normal distribution		2.020	4.736
Multibranched				
P13	Non-normal distribution	Kruskal–Wallis test	0.032	1.144
P21	Non-normal distribution		−0.107	1.309
P30	Non-normal distribution		−0.267	1.857
P50	Non-normal distribution		−0.154	0.398
P100	Non-normal distribution		−0.045	0.625
Atypical				
P13	Non-normal distribution	Kruskal–Wallis test	−0.150	0.713
P21	Non-normal distribution		−0.135	0.690
P30	Non-normal distribution		−0.611	1.579
P50	Not applicable		Not applicable	
P100	Non-normal distribution		−0.040	0.572
Figure 5*F*, iSPN				
Stubby				
P13	Normal distribution	Kruskal–Wallis test	32.329	40.112
P21	Non-normal distribution		31.046	43.463
P30	Normal distribution		34.412	42.389
P50	Normal distribution		33.412	41.381
P100	Normal distribution		34.637	39.443
Thin/Filopodial				
P13	Normal distribution	One-way ANOVA	40.414	48.479
P21	Normal distribution		38.564	48.200
P30	Normal distribution		34.343	43.229
P50	Normal distribution		36.824	42.283
P100	Normal distribution		34.603	40.100
Mushroom				
P13	Normal distribution	One-way ANOVA	9.046	16.662
P21	Normal distribution		12.614	21.038
P30	Normal distribution		15.105	22.990
P50	Normal distribution		14.417	22.040
P100	Normal distribution		19.207	26.732
Branched				
P13	Normal distribution	One-way ANOVA	2.419	8.748
P21	Normal distribution		0.658	4.182
P30	Normal distribution		2.002	5.530
P50	Normal distribution		2.462	5.799
P100	Normal distribution		1.196	2.832
Multibranched				
P13	Non-normal distribution	Kruskal–Wallis test	−0.603	1.978
P21	Non-normal distribution		−0.149	0.384
P30	Not applicable		Not applicable	
P50	Non-normal distribution		−0.375	1.239
P100	Non-normal distribution		−0.294	1.313
Atypical				
P13	Non-normal distribution	Kruskal–Wallis test	−0.263	0.680
P21	Not applicable		Not applicable	
P30	Not applicable		Not applicable	
P50	Non-normal distribution		−0.263	0.680
P100	Non-normal distribution		−0.132	0.362

### Data and materials availability

All data are available in the main text.

## Results

### Dendritic spines were pruned in dSPNs and iSPNs of the dorsolateral striatum after P30

We microinjected Cre-dependent *AAV9-EF1α-DIO-eYFP* reporter viruses into subregions of the striatum of P0–P2 striatum of *Drd1a-Cre* and *Adora2a-Cre* mice to label the dendritic spines of dSPNs and iSPNs, respectively. Microinjected brains were harvested for spine analysis at stages of early juvenile (P13), late juvenile (P21), early adolescence (P30), late adolescence (P50), and adulthood (P100).

**Figure 1.**
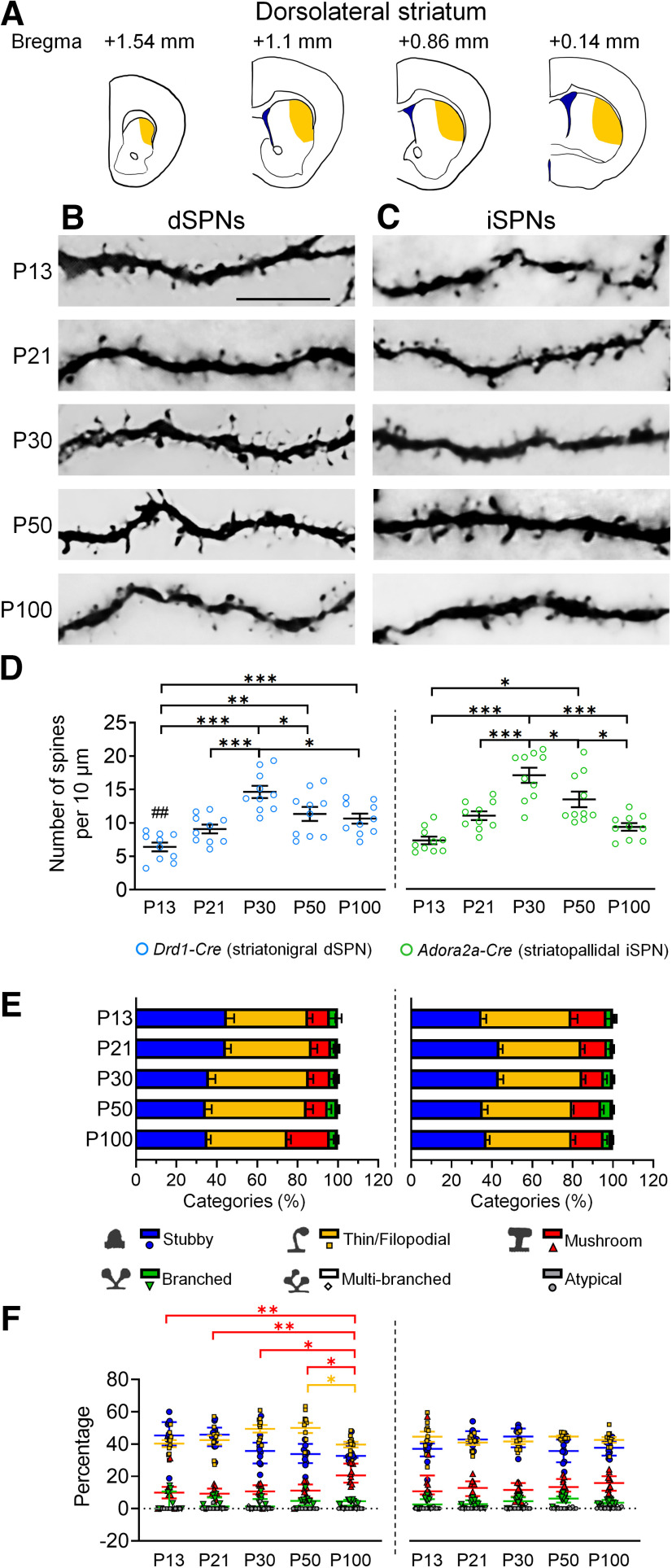
Development of dendritic spines in dSPNs and iSPNs of the dorsolateral striatum. ***A***, Schematic drawings show the regions of the dorsolateral striatum that are included for analysis. ***B***, ***C***, Immunohistochemistry of eYFP shows the development of eYFP-labeled dendritic spines in dSPNs and iSPNs, respectively, in the dorsolateral striatum (caudoputamen) of *Drd1-Cre* mice (***B***) and *Adora2a-Cre* mice (***C***). ***D***, Quantification of spine density. ***E***, Quantitative analysis of morphologic profiles of spines during development. ***F***, Dynamic changes of specific types of spines during development. * differences between ages. *p <* *0.05, **p <* *0.01, ***p <* *0.001. ^#^ differences between genotypes. ^##^*p *<* *0.01. Two-way ANOVA is used in ***D***. One-way ANOVA followed by Tukey’s HSD *post hoc* tests is used in ***F*** for the data that are normally distributed. Kruskal–Wallis one-way ANOVA followed by Dunn’s pairwise multiple comparisons tests are used in F for data that are not normally distributed. Data in ***D*** and ***E*** are mean ± SEM; Thin/Filopodial spine data in ***F*** are mean ± SEM; Stubby, Mushroom, Branched, Multi-branched and Atypical spine data in ***F*** are median ± interquatile range. *n *=* *10 cells from 2–3 mice/age. P, postnatal day. Scale bar: 10 μm.

Because the dorsolateral and dorsomedial parts of the striatum have distinct roles in the regulation of motor learning, habit formation and drug addiction (Thorn et al., 2010; Lipton et al., 2019), we investigated dendritic spinogenesis in the dorsolateral and dorsomedial striatum. In the dorsolateral striatum, the density of the spines in dSPNs increased to reach its peak at P30. Subsequently, the dendritic spines were significantly pruned between P30 and P50. At P100, the spine density returned to a level comparable to that of P21 (Fig. 1*A*,*B*,*D*).

Regarding the development of different types of dendritic spines in dSPNs, the mushroom type of spines gradually increased from P13 to P100 along with a reduction in thin/filopodial spines between P50 and P100 (Fig. 1*E*,*F*).

For iSPNs, the spine density progressively increased to reach the maximum level at P30. Spine pruning in the iSPN population subsequently occurred in P30–P100, which was longer than the pruning time window of P30–P50 in the dSPN population (Fig. 1*A*,*C*,*D*). However, with all spines, the percentage of each type of spine in iSPNs appeared to remain about the same level across developmental times (Fig. 1*E*,*F*).

### Dendritic spines were pruned in dSPNs and iSPNs of the dorsomedial striatum after P50

**Figure 2.**
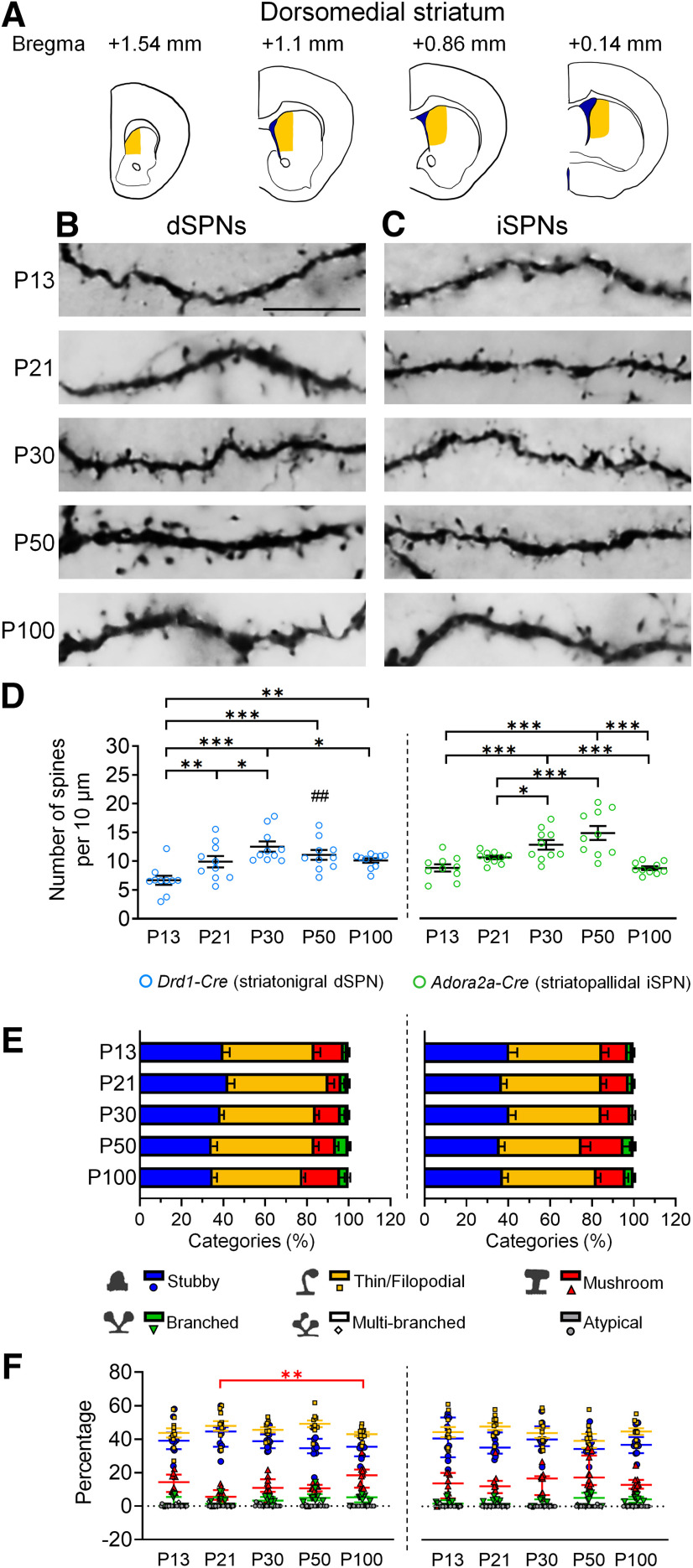
Development of dendritic spines in dSPNs and iSPNs of the dorsomedial striatum. ***A***, Schematic drawings show the regions of the dorsomedial striatum that are included for analysis. ***B***, ***C***, Immunohistochemistry of eYFP shows the development of eYFP-labeled dendritic spines in dSPNs and iSPNs, respectively, in the dorsomedial striatum (caudoputamen) of *Drd1-Cre* mice (***B***) and *Adora2a-Cre* mice (***C***). ***D***, Quantification of spine density. ***E***, Quantitative analysis of morphologic profiles of spines during development. ***F***, Dynamic changes of specific types of spines during development. * differences between ages. *p <* *0.05, **p <* *0.01, ***p <* *0.001. ^#^ differences between genotypes. ^##^*p *<* *0.01. Two-way ANOVA is used in ***D***. One-way ANOVA followed by Tukey’s HSD *post hoc* tests is used in ***F*** for the data that are normally distributed. Kruskal–Wallis one-way ANOVA followed by Dunn’s pairwise multiple comparisons tests are used in F for data that are not normally distributed. Data in ***D*** and ***E*** are mean ± SEM; Stubby and Thin/Filopodial spine data in ***F*** are mean ± SEM; Mushroom, Branched, Multi-branched and Atypical spine data in ***F*** are median ± interquatile range. *n *=* *10 cells from 2–3 mice/age. P, postnatal day. Scale bar: 10 μm.

In the dorsomedial striatum of dSPNs, the density of the spine reached its peak at P30, and the plateau level was maintained from P30 to P50. Subsequently, the dendritic spines underwent pruning between P50 and P100. By P100, the spine density dropped back to a level comparable to that of P21 (Fig. 2*A*,*B*,*D*).

For iSPNs, the spine density remained stable before P21 and progressively increased after P21. Compared with the peak of spines at P30 in the dSPN population, the spine density of iSPNs reached its maximum level later between P30–P50. By P100, the spine density of iSPNs fell back to a level comparable to that of P13–P21 (Fig. 2*A*,*C*,*D*). Interestingly, we found a higher spine density in the iSPNs at P13 and P50 compared with the dSPNs, which may result from a late-onset spine pruning of iSPNs. By P100 at adulthood, the spine density between dSPNs and iSPNs was similar.

As for temporal profiles of spine classification in the dorsomedial striatum, the proportion of mushroom spines were significantly increased in dSPNs by 1.87-fold at P100 (Fig. 2*E*,*F*). In contrast, no significant change in temporal profiles of spine classification was observed in iSPNs of the dorsomedial striatum (Fig. 2*E*,*F*).

Although the developmental trajectories of dendritic spines were different between the dorsolateral and dorsomedial SPNs, the spine densities of dSPNs and iSPNs were comparable between these two regions during postnatal development except at P30, the spiny density of iSPNs of the dorsomedial striatum was higher than that of the dorsolateral striatum (Table 2).

**Table 2:** Postnatal development of dendritic spines of striatonigral (dSPNs) and striatopallidal neurons (iSPNs) in the dorsolateral and dorsomedial striatum of male mice

	Striatonigral neurons/dSPNs		Striatopallidal neurons/iSPNs	
Age	DLS	DMS	DLS	DMS
P13	6.408 ± 0.650	6.687 ± 0.777	9.076 ± 0.719	8.786 ± 0.639
P21	9.087 ± 0.658	9.921 ± 0.992	11.007 ± 0.659	10.606 ± 0.319
P30	14.650 ± 0.917	12.539 ± 0.893	17.079 ± 1.129**	12.803 ± 0.844
P50	11.351 ± 1.051	11.351 ± 1.051	13.443 ± 1.169	14.848 ± 1.124
P100	10.646 ± 0.744	10.115 ± 0.399	9.318 ± 0.573	8.741 ± 0.320

The numbers indicate the density of dendritic spines (mean ± SEM) on different postnatal days (P).

**p <* *0.01. DLS, dorsolateral striatum; DMS, dorsomedial striatum.

### Dendritic spines were preferentially pruned in dSPNs but not in iSPNs in the shell region of NAc

It has been shown that dSPN and iSPN populations in the medial and lateral parts of the NAc shell serve different functions, e.g., dSPNs and iSPNs in the NAc shell differentially regulate reward and aversion (Yang et al., 2018; Yao et al., 2021). We investigated spinogenesis in the medial and lateral parts of the NAc shell.

**Figure 3.**
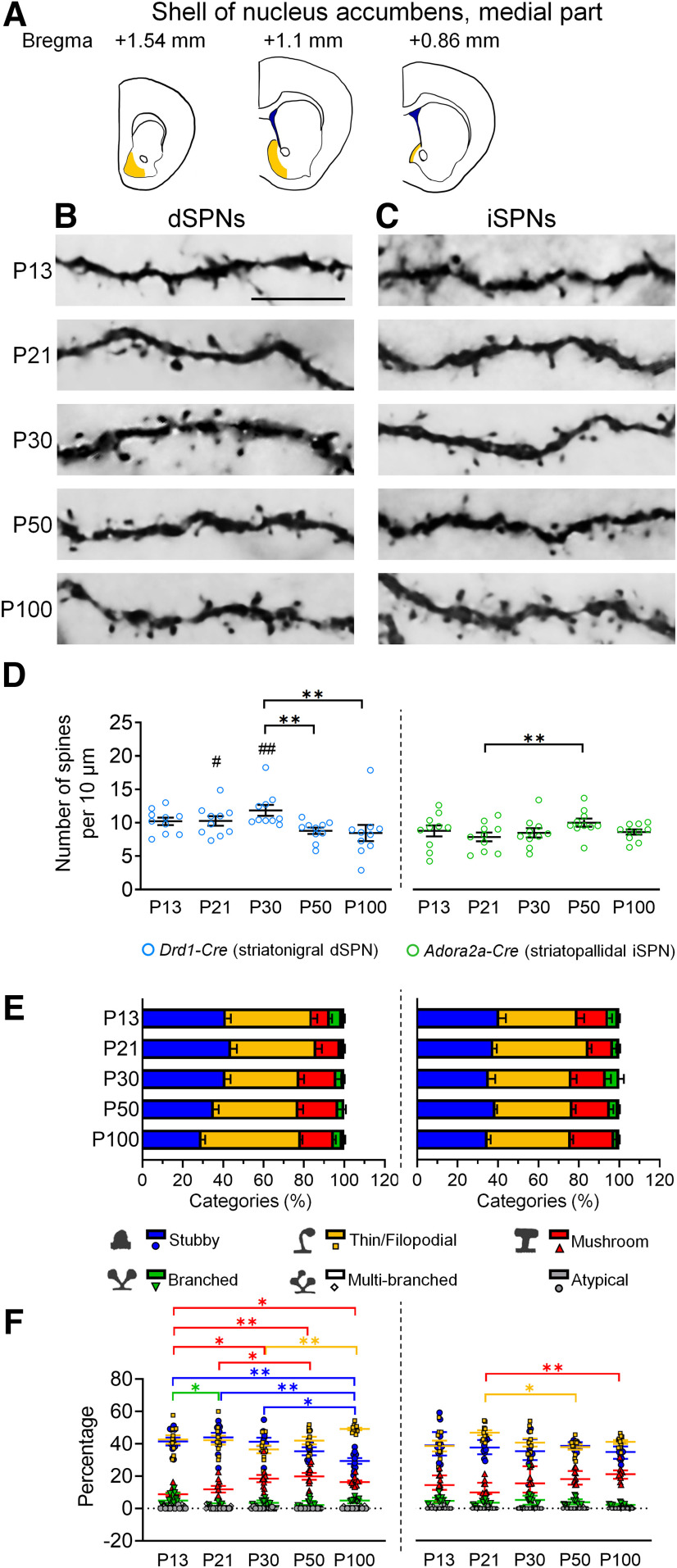
Development of dendritic spines in dSPNs and iSPNs of the medial shell of the nucleus accumbens. ***A***, Schematic drawings show the regions of the medial shell of the nucleus accumbens that are included for analysis. ***B***, ***C***, Immunohistochemistry of eYFP shows the development of eYFP-labeled dendritic spines in dSPNs and iSPNs, respectively, in the medial shell of the nucleus accumbens of *Drd1-Cre* mice (***B***) and *Adora2a-Cre* mice (***C***). ***D***, Quantification of spine density. ***E***, Quantitative analysis of morphologic profiles of spines during development. ***F***, Dynamic changes of specific types of spines during development. * differences between ages. *p <* *0.05, **p <* *0.01, ***p <* *0.001. ^#^ differences between genotypes. ^#^*p *<* *0.05. ^##^*p *<* *0.01. Two-way ANOVA is used in ***D***. One-way ANOVA followed by Tukey’s HSD *post hoc* tests is used in ***F*** for the data that are normally distributed. Kruskal–Wallis one-way ANOVA followed by Dunn’s pairwise multiple comparisons tests were used in F for data that are not normally distributed. Data in ***D*** and ***E*** are mean ± SEM; Stubby, Thin/Filopodial and Mushroom spine data in ***F*** are mean ± SEM; Branched, Multi-branched and Atypical spine data in ***F*** are median ± interquatile range. *n *=* *10 cells from 2–3 mice/age. P, postnatal day. Scale bar: 10 μm.

In the medial part of the NAc shell, the spine density of dSPNs was already high at P13, and a high level was maintained until P30. Marked spine pruning was found between P30 and P50. A trend of decreasing spine density was observed at P30–P100 (Fig. 3*A*,*B*,*D*). In contrast to dSPNs, no prominent spine pruning was found in iSPNs from P13 to P100, although there was a moderately increasing trend from P21 to P50 (Fig. 3*A*,*C*,*D*).

**Figure 4.**
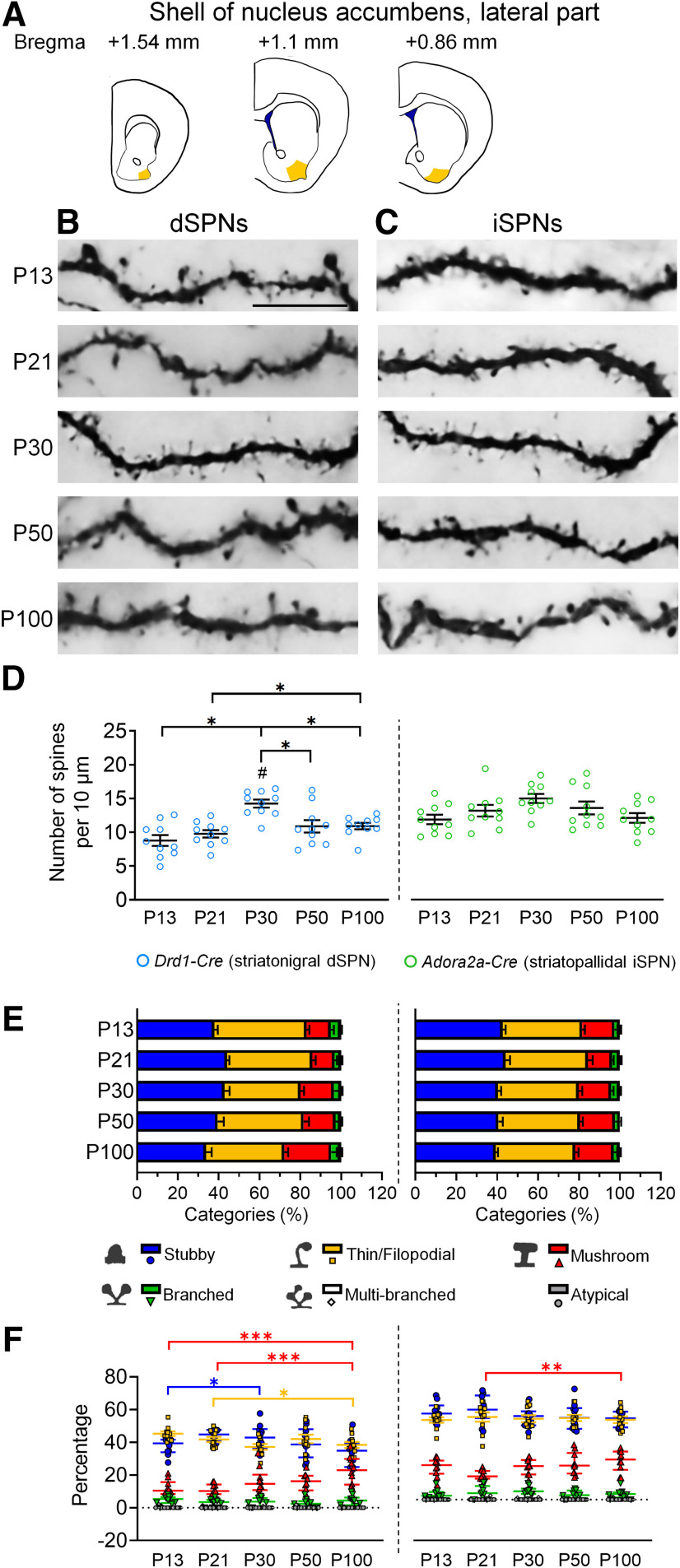
Development of dendritic spines in dSPNs and iSPNs of the lateral shell of the nucleus accumbens. ***A***, Schematic drawings show the regions of the lateral shell of the nucleus accumbens that are included for analysis. ***B***, ***C***, Immunohistochemistry of eYFP shows the development of eYFP-labeled dendritic spines in dSPNs and iSPNs, respectively, in the lateral shell of the nucleus accumbens of *Drd1-Cre* mice (***B***) and *Adora2a-Cre* mice (***C***). ***D***, Quantification of spine density. ***E***, Quantitative analysis of morphologic profiles of spines during development. ***F***, Dynamic changes of specific types of spines during development. * differences between ages. *p <* *0.05, **p <* *0.01, ***p <* *0.001. ^#^ differences between genotypes. ^#^*p *<* *0.05. Two-way ANOVA is used in ***D***. One-way ANOVA followed by Tukey’s HSD *post hoc* tests is used in ***F*** for the data that are normally distributed. Kruskal–Wallis one-way ANOVA followed by Dunn’s pairwise multiple comparisons tests are used in F for data that are not normally distributed. Data in ***D*** and ***E*** are mean ± SEM; Stubby, Thin/Filopodial, Mushroom, Branched spine data of dSPN and Stubby, Mushroom, Branched spine data of iSPN in ***F*** are mean ± SEM; Multi-branched, Atypical spine data of dSPN and Thin/Filopodial, Multi-branched, Atypical spine data of iSPN in ***F*** are median ± interquatile range. *n *=* *10 cells from 2–4 mice/age. P, postnatal day. Scale bar: 10 μm.

In the lateral part of the NAc shell, the spine density of dSPNs reached its peak at P30, and spine pruning was observed between P30 and P50. At P100, the spine density was slightly higher than that at P13–P21 (Fig. 4*A*,*B*,*D*). For iSPNs, no substantial changes in the spine density were found in the lateral part of the NAc shell from P13 to P100 (Fig. 4*A*,*C*,*D*).

Regarding temporal profiles of morphologic changes of dendritic spines, progressively increasing proportions of mushroom spines were found in both dSPN and iSPN of lateral and medial parts of the NAc shell (Figs. 3*E*,*F*, 4*E*,*F*). Decreasing levels of stubby or thin/filopodial spines were also found in dSPNs of medial and lateral parts of the NAc shell and iSPNs of the medial part of the NAc shell, although there was a slight increase in thin/filopodial spines between P30 and P100 in dSPNs of medial NAc shell (Figs. 3*E*,*F*, 4*E*,*F*). These results suggest a progressive maturation of dendritic spines in dSPNs and iSPNs of NAc shell from immature to mature types during postnatal development.

### Spine pruning progressively occurred in dSPNs and iSPNs in the core region of NAc

**Figure 5.**
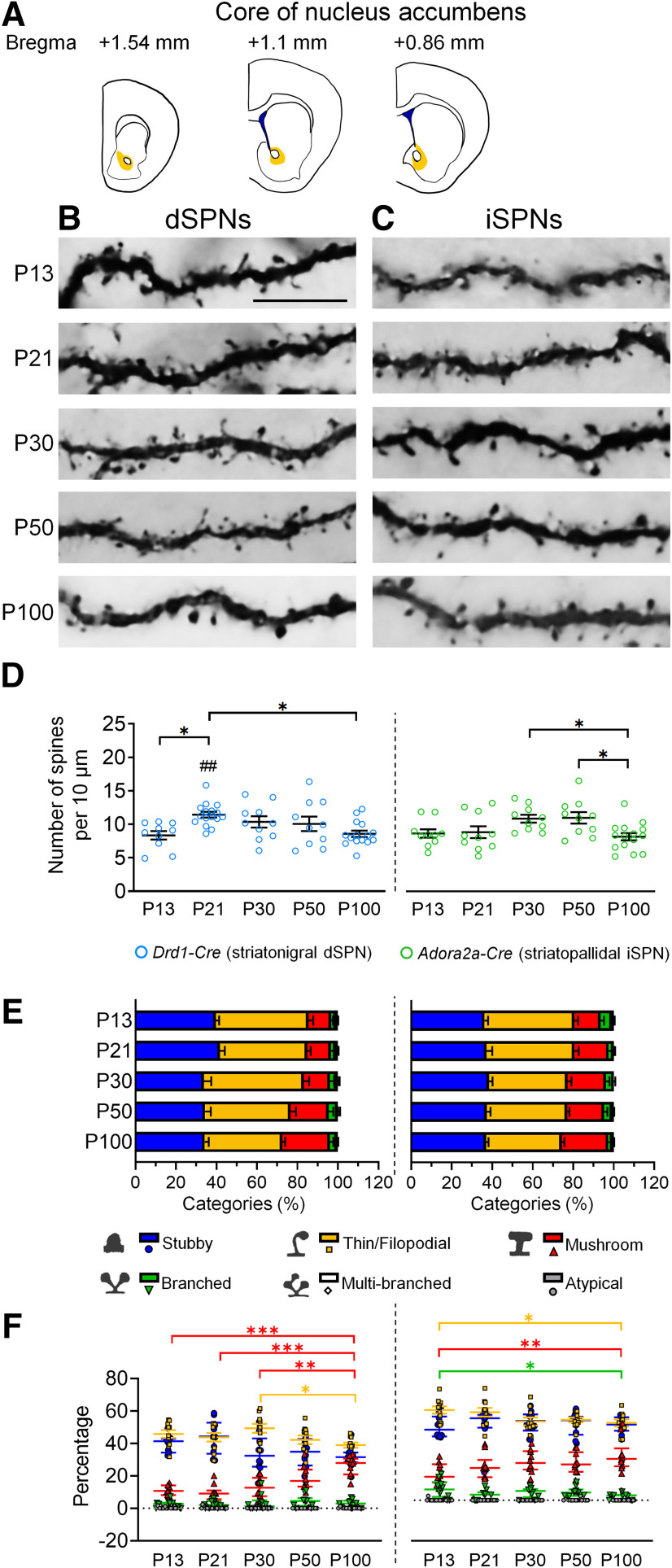
Development of dendritic spines in dSPNs and iSPNs of the core of the nucleus accumbens. ***A***, Schematic drawings show the regions of the core of the nucleus accumbens that are included for analysis. ***B***, ***C***, Immunohistochemistry of eYFP shows the development of eYFP-labeled dendritic spines in dSPNs and iSPNs, respectively, in the core of the nucleus accumbens of *Drd1-Cre* mice (***B***) and *Adora2a-Cre* mice (***C***). ***D***, Quantification of spine density. ***E***, Quantitative analysis of morphologic profiles of spines during development. ***F***, Dynamic changes of specific types of spines during development. * differences between ages. *p <* *0.05, **p <* *0.01, ***p <* *0.001. ^#^ differences between genotypes. ^##^*p *< 0.01. Two-way ANOVA is used in ***D***. One-way ANOVA followed by Tukey’s HSD *post hoc* tests are used in F for the data that are normally distributed. Kruskal–Wallis one-way ANOVA followed by Dunn’s pairwise multiple comparisons tests are used in ***F*** for data that are not normally distributed. Data in ***D*** and ***E*** are mean ± SEM; Stubby, Thin/Filopodial, Mushroom spine data of dSPN and Thin/Filopodial, Mushroom, Branched spine data of iSPN in ***F*** are mean ± SEM; Branched, Multi-branched, Atypical spine data of dSPN and Stubby, Multi-branched, Atypical spine data of iSPN in ***F*** are median ± interquatile range. *n *=* *10–16 cells from 2–3 mice/age. P, postnatal day. Scale bar: 10 μm.

In the NAc core, the maximum level of spine density of dSPNs was observed at P21. Subsequently, spine density decreased until P100, at which time the dendritic spines were at a level comparable to that of P13 (Fig. 5*A*,*B*,*D*). For iSPNs, spine density reached its peak level at P30 and P50 and was then gradually decreased from P50 to P100 (Fig. 5*A*,*C*,*D*).

For morphologic analysis, thin/filopodial spines were reduced after P30, accompanied by a gradual increase of mushroom spines in dSPNs throughout postnatal development (Fig. 5*E*,*F*). As for iSPNs, a gradually increased proportion of mushroom spines was found from P13 to P100. Similar to dSPNs, the proportion of thin/filopodial spines in iSPNs was lower at P100 compared with that of P13 (Fig. 5*E*,*F*).

Among the different regions of the NAc, the spine density of dSPNs in the core of NAc core was lower than that of dSPNs in the lateral part of NAc shell at P30 and P100 (Table 3), which was consistent with the prominent pruning found in the core of NAc after P21 (Fig. 5*D*). Furthermore, the spine density of iSPNs in the medial part of NAc shell was lowest at P21 compared with other regions of NAc (Table 3; Fig. 3*D*).

**Table 3:** Postnatal development of dendritic spines of striatonigral (dSPNs) and striatopallidal neurons (iSPNs) in subregions of the nucleus accumbens of male mice

Age	NAcc	NAcsLat	NAcsMed
Striatonigral neurons/dSPNs			
P13	8.356 ± 0.634	8.780 ± 0.803	10.222 ± 0.562
P21	11.492 ± 0.685	9.772 ± 0.546	10.265 ± 0.712
P30	10.359 ± 0.849^#^	14.250 ± 0.596	11.852 ± 0.822
P50	10.068 ± 1.087	10.884 ± 0.919	8.797 ± 0.486
P100	8.700 ± 0.675^#^	10.923 ± 0.474	8.461 ± 1.215^#^
Striatopallidal neurons/iSPNs			
P13	8.657 ± 0.616	9.501 ± 0.557	8.792 ± 0.825
P21	8.843 ± 0.878*	10.539 ± 0.681**	7.876 ± 0.659
P30	10.860 ± 0.587	11.990 ± 0.536	8.495 ± 0.699
P50	10.979 ± 0.857	10.861 ± 0.764	10.019 ± 0.613
P100	8.137 ± 0.816	9.681 ± 0.575	8.618 ± 0.395

The numbers indicate the density of dendritic spines (mean ± SEM) on different postnatal days (P). ^#^ versus NAcsLat, ^#^*p *<* *0.05. * versus NAcsMed, *p <* *0.05, **p <* *0.01. NAcc: nucleus accumbens core; NAcsLat: lateral part of nucleus accumbens shell; NAcsMed: medial part of nucleus accumbens shell.

## Discussion

We investigated the developmental trajectories of dendritic spines of dSPN and iSPN populations in the caudoputamen and NAc. We took advantage of the Cre/LoxP system and viral targeting strategies to label specific cell types of SPNs. With the aid of eYFP immunohistochemistry, it allows us to identify the protrusion of eYFP-positive dendritic spines of SPNs, although the resolution was limited to resolve the morphologic detail of individual spines. Our study complements previous reports and provides new information on spine formation in striatal neurons at three levels. First, we analyzed spinogenesis in subregions of the striatal complex, including the medial and lateral parts of the caudoputamen (dorsal striatum), and the core and shell regions of the NAc (ventral striatum). Second, in each striatal subregion, we provide a cell-type characterization of spinogenesis in dSPN and iSPN populations. Third, by analyzing multiple time points in postnatal stages, including early juvenile at P13, late juvenile at P21, early adolescence at P30, late adolescence at P50, and adulthood at P100, we delineated the developmental trajectories of dSPN and iSPN populations in each striatal subregion.

In general, we found that the dendritic spines of dSPNs and iSPNs progressively increased at the early stages of postnatal development, followed by prominent spine pruning beginning from adolescence in both the dorsal and ventral striatum. The developmental maturation of dendritic spines in dSPNs and iSPNs, however, follow different spatiotemporal trajectories in the dorsal and ventral striatum, implicating cell type-specific maturation of striatal circuits.

### Developmental maturation of dendritic spines in the caudoputamen of striatal circuitry

Dendritic spines are presumably the loci of excitatory synapses. SPNs receive excitatory inputs from the cerebral cortex and the thalamus. Despite some discrepancies among different species, corticostriatal and thalamostriatal axonal terminals preferentially innervate, respectively, the head and shaft of spines (Smith et al., 2004; Liu et al., 2011). Previous studies have characterized spinogenesis in the dorsal striatum in early postnatal development. Immature dendritic spines of striatal neurons occur as early as P6 (Lee and Sawatari, 2011). Mature spines appear in striatal neurons at P8-P9 (Lee and Sawatari, 2011; Chen et al., 2016), followed by extensive growth of spines at P10–P28 (Tepper et al., 1998; Uryu et al., 1999; Peixoto et al., 2016; Krajeski et al., 2019). Electron microscopic study has reported synaptic pruning in the striatum from P18 to adulthood (Uryu et al., 1999). These previous studies, however, do not provide information on spine development and maturation in specific cell types in the striatum.

In the present study, we found that in the caudoputamen, the density of spines in dSPNs and iSPNs progressively increased in the early stages of the postnatal period before P30, followed by prominent spine pruning in both medial and lateral parts of the caudoputamen after P30–P50 onwards. The findings of progressive increases in SPN spines before P30 are likely to reflect the increasing innervations of SPNs by the cortex and thalamus (Krajeski et al., 2019). Consistent with these results, previous electrophysiological study with morphologic analysis shows that a gradual increase in dendritic spines occurs parallelly in dSPNs and iSPNs at P3–P28, although this study does not have spine data after P35 (Krajeski et al., 2019).

We further found a difference in the trajectory of spine pruning in dSPNs and iSPNs. For the dSPN population, the time window of spine pruning occurred after P30, which was earlier than the iSPN population in which spine pruning occurred after P50. The cellular mechanisms underlying the differential regulation of spine pruning time windows in dSPNs and iSPNs are not yet known. Distinct intrinsic electrophysiological properties and biased inhibitory inputs to dSPN and iSPN populations after the second postnatal week may be involved in the differential regulation of spinogenesis (Krajeski et al., 2019).

The developmental maturation of dendritic spines is likely to reflect the functional maturation of excitatory synapses. Previous electrophysiological and optogenetic study has demonstrated that progressively increased amplitudes of optically evoked excitatory postsynaptic currents in corticostriatal circuits are correlated with developmental increases in the ratio of AMPA/NMDA currents and dendritic spine density of SPNs in the first postnatal month period (Peixoto et al., 2016), suggesting a strong functional interaction between the developing cortex and striatum during corticostriatal circuit wiring.

### Developmental maturation of dendritic spines in the nucleus accumbens of striatal circuitry

Despite that neural plasticity of dendritic spines of SPNs is well characterized in the NAc under different behavioral states, including reward learning, drug addiction and chronic social defeat-induced stress (LaPlant et al., 2010; Fox et al., 2020; Iino et al., 2020; Thompson et al., 2021), the developmental trajectory of spinogenesis in the NAc has not yet been fully characterized. Previous studies have reported that spine density is stably maintained in SPNs of the NAc core of the rat brain at P21–P70 (Bringas et al., 2013; Tendilla-Beltrán et al., 2016).

Our present study found a dynamic profile of spine development and maturation in dSPNs and iSPNs of the NAc. The spine density of dSPNs and iSPNs in the NAc core reached its peak level at P21 and P30, respectively, and subsequently decreased in postnatal periods. Microglia plays an important role in spine pruning (Paolicelli et al., 2011). Mallya et al., have reported that microglia-mediated engulfment of presynaptic terminals and postsynaptic dendritic spines is involved in the elimination of synapses in the prefrontal cortex during adolescence (Mallya et al., 2019). Interestingly, a marked increase in the proliferation of microglia occurs in the NAc during the third postnatal week (Hope et al., 2020). Given microglia-mediated phagocytic activity to prune synapses evident in the prefrontal cortex in postnatal development (Mallya et al., 2019), microglia-mediated synaptic pruning may be involved in shaping the maturation patterns of spines in dSPNs and iSPNs that we observed in the NAc. Such microglia-mediated synaptic modification conceivably may have a behavioral impact. For example, microglia and complement-mediated phagocytic activity have been shown to shape NAc development by eliminating dopamine D1R, which impacts the development of social behavior in adolescent rats (Kopec et al., 2018). Interestingly, microglia with distinct physiological characteristics emerge in different regions of basal ganglia during the second postnatal week (De Biase et al., 2017). Because microglia are implicated in the differential responses of excitatory postsynaptic currents in dSPNs and iSPNs of the dorsal striatum in adult mice (Hayes et al., 2022), it will be of interest to see whether region-specific microglia play a role in determining differential trajectories of spine maturation in SPNs of the caudoputamen and NAc.

### Activity-dependent regulation of the development and maturation of spines/synapses of SPNs in striatal circuitry

Sensory inputs and learning-related experiences can induce neuroplastic changes in spine formation and elimination (Berry and Nedivi, 2017), indicating that neuronal activity plays an important role in spinogenesis/synaptogenesis. The striatum receives three major afferents from the cerebral cortex, thalamus, and ventral midbrain. SPNs integrate glutamatergic inputs through corticostriatal and thalamostriatal pathways. The activities of glutamatergic inputs are influential in spinogenesis and synaptic wiring during development. It has been proposed that the balanced activity of dSPN and iSPN pathways controls glutamatergic synaptogenesis/spinogensis via recurrent closed loops of cortico-basal ganglia-thalamus circuits (Kozorovitskiy et al., 2012). It is yet unknown whether thalamostriatal activity could affect synaptogenesis/spinogensis of SPNs. Despite that the percentages of corticostriatal and thalamostriatal synapses in dSPNs and iSPNs are varied among different studies (Doig et al., 2010; Lei et al., 2013), it has been shown that repetitive stimulations of corticostriatal and thalamostriatal pathways, respectively, increase and decrease postsynaptic depolarization in dSPNs and iSPNs (Ding et al., 2008). Given the nature of activity-dependent regulation of spine/synapse formation, it will be of great interest to look into the possibility of how corticostriatal and thalamostriatal glutamatergic inputs are integrated and as a consequence impact the trajectories of spine maturation in dSPNs and iSPNs that we observed in the present study.

Synaptic plasticity, including long-term potentiation (LTP) and long-term depression (LTD) that occurs in glutamatergic synapses of SPNs, may play a role in sculpting dendritic spines during development. In the rat striatum, LTP and LTD can occur in striatal neurons in early postnatal stages of P12–P15 (Partridge et al., 2000). However, in the mouse striatum, Maltese et al., have reported that LTP and LTD cannot be stably induced, respectively, before P24 and P28 (Maltese et al., 2018). Furthermore, the endocannabinoid receptor (CB1R)-mediated LTD can only be induced in corticostriatal synapses, but not in thalamostriatal synapses in adult brains (Wu et al., 2015). Intriguingly, the CB1R expression in corticostriatal terminals undergoes prominent downregulation after P28 in both the cortex and striatum (Van Waes et al., 2012), which coincides with the period of spine pruning in SPNs of the caudoputamen. Given the ability of LTP and LTD in shaping spine formation and elimination, it will be of interest to see whether neural plasticity mechanisms of LTP and LTD may participate in the spine/synapse maturation of dSPNs and iSPNs.

Dopamine inputs from the ventral midbrain are important for striatal development and maturation. Dopamine increases dSPN activity but decreases iSPN activity (Nishi et al., 2000), which may, in turn, regulate spinogenesis. It has been shown that chemogenetic inhibition of dSPNs and iSPNs during P8–P14, respectively, decreases and increases dendritic spines (Kozorovitskiy et al., 2012). Dopamine is known to be involved in neural plasticity of striatal LTP, LTD and spinogenesis (Martel and Galvan, 2022), and activation of D1R and D2R promotes, respectively, LTP and LTD in striatal glutamatergic synapses (Shen et al., 2008; Higley and Sabatini, 2010). Interestingly, the spine formation of SPNs can be modulated by D1R and A2aR signalings in P8–P13 (Kozorovitskiy et al., 2015). The co-activation of D1R and A2aR increases the spines of iSPNs (Kozorovitskiy et al., 2015), whereas genetic deletion of D1R decreases the spines of dSPNs and iSPNs (Suarez et al., 2020).

Intrinsic inputs from local interneurons may sculpt the trajectory of SPN spinogenesis. Late-onset maturation of local inhibitory interneurons may contribute to shaping the excitability of SPNs by increasing inhibitory inputs that could result in spine pruning (Krajeski et al., 2019). Notably, as cholinergic interneurons can modulate corticostriatal LTD (Wang et al., 2006), such cholinergic interneuron-modulated LTD activity may, in turn, regulate spinogenesis.

### Dysregulation of synaptogenesis and spinogenesis in neuropsychiatric diseases

Developmental modification of dendritic spines is critical to the maturation of precise neural networks. Defective development and plasticity of dendritic spines are associated with the pathophysiology of neuropsychiatric disorders (Forrest et al., 2018). Aberrant synaptic pruning had been reported in cortical pyramidal neurons in patients with autism spectrum disorder (ASD) (Hutsler and Zhang, 2010) and animal models of ASD (Pan et al., 2010; Jiang et al., 2013; Isshiki et al., 2014). Abnormal spinogenesis occurs in striatal neurons of ASD mouse models (Peça et al., 2011; Chen et al., 2016), e.g., genetic mutation of the autism-risk gene *Shank3* results in a selective decrease in spine density of iSPNs but not dSPNs (Wang et al., 2017). Furthermore, the re-introduction of *Shank3* gene expression in adult striatal neurons rescues dendritic spine deficits and behavioral abnormalities in *Shank3* knock-out mice (Mei et al., 2016). Excessive spine pruning and spine dysgenesis have also been reported in patients with schizophrenia (Feinberg, 1982; Roberts et al., 1996; MacDonald et al., 2017; McKinney et al., 2019; Onwordi et al., 2020), in which excessive synaptic pruning may be mediated by microglia (Sellgren et al., 2019). These clinical and preclinical studies highlight the importance of understanding synaptogenesis and spinogenesis in postnatal brains during maturation. Our current study, therefore, provides a basic reference to neurodevelopmental diseases in which spinogenesis and synaptogenesis are affected in striatal neurons.

### Limitations and issues for further work

Our study is subject to limitations. We have only focused on the characterization of spine development. We did not investigate the potential role of cortical and thalamic excitatory inputs in the regulation of the development and maturation of dendritic spines in dSPNs and iSPNs. This is an interesting and important question, given the key role of neuronal activity in determining spine/synapse structure and function (Segal and Andersen, 2000; Martel and Galvan, 2022). The whole-brain mapping study has shown that the direct dSPN and indirect iSPN pathways receive overlapping and yet differential inputs from the cortex and thalamus (Wall et al., 2013; Guo et al., 2015). Network connectivity coupled with intrinsic neurochemical and molecular differences between dSPNs and iSPNs may underlie differential development and maturation of spines in the caudoputamen and NAc. The resulting precise synaptic wiring through spine/synapse maturation ensures proper circuit functions in the basal ganglia.

Despite these and other limitations, our study delineates the trajectories of spine maturation in dSPNs and iSPNs in the motor circuitry-related caudoputamen and the limbic circuitry-associated NAc of the striatal complex. Our study provides a basic reference for future studies on the maturation of striatal circuitry and neurologic diseases related to basal ganglia.

## Synthesis

Reviewing Editor: Christophe Bernard, INSERM & Institut de Neurosciences des Systèmes Decisions are customarily a result of the Reviewing Editor and the peer reviewers coming together and discussing their recommendations until a consensus is reached. When revisions are invited, a fact-based synthesis statement explaining their decision and outlining what is needed to prepare a revision will be listed below. The following reviewer(s) agreed to reveal their identity: Kenneth Campbell.

Your reviewers and I agree that some attempt should be made in the discussion to evaluate those results in the context of overall maturation of striatal neuronal circuitry.

We also regret that you did not try to assess the striatal afferent inputs in relation to the spine development/maturation. The available tissue would be sufficient to perform such experiments. But we also understand that you may not wish to include such analysis. This is up to you.

In conclusion, if you decide not to address the previous issue, just expand the discussion as requested.
